# State-dependent repetitive transcranial magnetic stimulation in disorders of consciousness

**DOI:** 10.3389/fnhum.2026.1840746

**Published:** 2026-05-29

**Authors:** Zhong Li, Jing Fu, Jianlin Pu, Yu Yin, Zhen Han, Hongpeng Liu, Yadong Liu, Xuesong Gai, Li Li

**Affiliations:** 1 Second Clinical Medical College, Yunnan University of Chinese Medicine, Kunming, Yunnan, China; 2 School of Traditional Dai Medicine, West Yunnan University of Applied Sciences, Xishuangbanna, China; 3 Department of Rehabilitation Medicine, The First People’s Hospital of Yunnan Province, Kunming, Yunnan, China; 4 Department of Emergency Trauma Surgery, The First People’s Hospital of Yunnan Province, Kunming, Yunnan, China

**Keywords:** disorders of consciousness, minimally conscious state, repetitive transcranial magnetic stimulation, residual consciousness, TMS-EEG, vegetative state/unresponsive wakefulness syndrome

## Abstract

Disorders of consciousness (DOCs), including coma, vegetative state/unresponsive wakefulness syndrome (VS/UWS), and minimally conscious state (MCS), remain among the most difficult conditions to manage in neurorehabilitation. Repetitive transcranial magnetic stimulation (rTMS) has emerged as a promising neuromodulatory approach, yet its clinical effects appear to vary substantially across DOC subtypes, and the basis of this response gradient remains incompletely understood. In this narrative review, we used a structured literature-search approach and prioritized randomized or sham-controlled clinical studies, controlled observational studies, and mechanistic studies integrating rTMS with neuroimaging and/or electrophysiological measures to synthesize current evidence on therapeutic efficacy and putative mechanisms across DOC subtypes. Available data, interpreted in light of unequal evidence strength across subtypes, indicate that rTMS most consistently improves Coma Recovery Scale–Revised (CRS-R) scores in patients with MCS, shows heterogeneous effects in VS/UWS, and lacks sufficient therapeutic evidence in coma. Converging neurophysiological findings, although not equivalent to therapeutic efficacy, further suggest that coma and VS/UWS are often characterized by reduced thalamo-cortical reactivity and limited plastic/metaplastic capacity, whereas MCS more commonly retains partially preserved frontoparietal networks that may provide a plausible substrate for therapeutic rTMS testing. Taken together, these heterogeneous findings may be organized through a state-dependent, hypothesis-generating perspective on rTMS application in DOC, while the uneven evidence base remains the primary interpretive constraint. Within this secondary conceptual heuristic, rTMS in MCS may be better understood as a form of network fine-tuning, VS/UWS as an experimental network rebooting hypothesis, and coma primarily as an evidence gap in which TMS-EEG probing may inform future therapeutic studies rather than current treatment guidance. Rather than serving as a validated treatment algorithm, this subtype-, population-, and biomarker-informed framework is intended to organize current evidence, clarify evidence gaps, identify candidate readiness-related biomarkers, and inform future hypothesis-driven studies of biomarker-informed neuromodulation and residual consciousness-related stratification in DOC.

## 1 Introduction

Disorders of consciousness (DOCs), a spectrum of conditions ranging from coma to the minimally conscious state (MCS), represent a major challenge in modern neurorehabilitation (Giacino et al., 2018). Owing to diverse etiologies, including traumatic brain injury, hypoxic–ischemic injury, stroke, infection, toxic-metabolic encephalopathy, and other acquired brain injuries, DOC poses a significant challenge in clinical neurology (Lippert and Guggisberg, 2023). Accordingly, age, etiology, time since injury, resource setting, and access to specialized neurorehabilitation should be considered when interpreting diagnostic TMS markers and therapeutic rTMS responses. The clinical burden is substantial and is compounded by diagnostic uncertainty, with misdiagnosis rates for patients in a VS reported to be as high as 34% (Wade, 2018). This uncertainty is directly relevant to rTMS interpretation, because apparent subtype-dependent responsiveness may partly reflect imperfect baseline classification, fluctuating arousal, or unrecognized covert consciousness rather than true differences in stimulation efficacy. Moreover, the prognosis for correctly diagnosed patients remains limited, with high rates of long-term disability and mortality. Illustrating this research problem, one large observational study reported that DOC patients showed limited improvement, underscoring the limitations of current standard care (Romaniello et al., 2016). For every study demonstrating a positive response, another reports minimal or no effect, leaving clinicians and researchers facing conflicting results and therapeutic uncertainty.

Repetitive transcranial magnetic stimulation (rTMS) is a noninvasive neuromodulation technique that applies trains of magnetic pulses to the scalp, inducing focal electric currents in the underlying cortex (Pell et al., 2011). This process modulates cortical excitability and network activity, providing a plausible therapeutic rationale for neurorehabilitation, although current DOC evidence remains heterogeneous and predominantly derived from small-scale studies (Lefaucheur, 2019). Available trials and meta-analyses suggest that rTMS may yield clinically meaningful improvements in CRS-R scores in selected DOC populations, although the magnitude and reproducibility of these effects remain influenced by DOC subtype, etiology, age, disease stage, and study design (O'Neal et al., 2021; Yang et al., 2023). Beyond its therapeutic application, the broader technology of transcranial magnetic stimulation (TMS), when combined with electroencephalography (TMS-EEG), serves as a powerful diagnostic tool and can help detect covert consciousness in behaviorally unresponsive patients (Arai et al., 2021). In this review, evidence of residual reactivity, connectivity, or covert network capacity is treated as diagnostic, prognostic, or stratification evidence unless it is accompanied by clinically meaningful outcome changes after therapeutic rTMS. Such biomarkers are especially important for reducing diagnostic uncertainty and identifying patients with covert motor dissociation or hidden command-following who may be behaviorally classified as VS/UWS but neurophysiologically resemble higher-readiness states. In this review, we use “residual consciousness” in a constrained, multi-level sense. At the behavioral level, it refers to reproducible clinical signs of awareness detected by standardized assessment, such as CRS-R-defined MCS − or MCS + behaviors. At the electrophysiological or neuroimaging level, it refers to evidence of covert command-following, cognitive motor dissociation, long-range cortical propagation, or preserved network connectivity detected by EEG, TMS-EEG, fMRI, or PET despite limited behavior. At the theoretical level, it refers more cautiously to residual network capacity or plastic/metaplastic readiness that may support future therapeutic testing. These levels should not be treated as interchangeable: network reactivity or plasticity reserve may indicate residual capacity, but it does not by itself prove conscious awareness or therapeutic recovery. However, available studies suggest a provisional therapeutic gradient: responses appear more consistent in MCS, mixed in VS/UWS, and insufficiently established in coma. This gradient should be interpreted cautiously because it may reflect a combination of diagnostic state, residual network integrity, and study-design bias: VS/UWS includes sham-controlled studies with both positive and negative findings, whereas MCS evidence still relies partly on small mixed-subtype cohorts, uncontrolled studies, and case reports.

Against this heterogeneous and uneven evidence base, we interpret the provisional response gradient through a cautious, hypothesis-generating lens. In VS/UWS, the therapeutic challenge may be conceptualized as “network rebooting,” namely the attempt to re-establish basic cortical reactivity and distributed responsiveness from a state of marked network inertia. In coma, however, this concept should be treated more cautiously as a future-oriented probing hypothesis, because therapeutic targets, parameters, and responder profiles remain undefined. In contrast, in MCS, the challenge may be closer to “network fine-tuning,” which asks whether stimulation can improve the efficiency and integration of partially preserved but dysfunctional awareness-related networks. This framework should not be interpreted as a validated clinical rule. Rather, after acknowledging the heterogeneity and unequal strength of the evidence, it is intended only as a conceptual heuristic that links clinical state, residual network integrity, biomarker evidence, and stimulation goals into a testable structure for future studies.

### 1.1 Review approach and evidence selection

This article was designed as a narrative review rather than a systematic review or meta-analysis. To improve transparency, we conducted a structured literature search in PubMed/MEDLINE, Web of Science, Embase, Scopus, and the Cochrane Library from database inception to March 2026, supplemented by citation tracking of key reviews and reference lists. Search terms combined DOC-related terms (“disorders of consciousness,” “coma,” “vegetative state,” “unresponsive wakefulness syndrome,” “minimally conscious state,” “MCS,” “VS/UWS”) with stimulation-related terms (“transcranial magnetic stimulation,” “TMS,” “rTMS,” “theta-burst stimulation,” “TBS,” “iTBS”) and biomarker/outcome-related terms (“TMS-EEG,” “EEG,” “fMRI,” “PET,” “neuroimaging,” “connectivity,” “CRS-R,” and “recovery”). We prioritized peer-reviewed human studies directly evaluating therapeutic rTMS or patterned stimulation in DOC, especially randomized, sham-controlled, crossover, or controlled studies with clinical outcomes and/or electrophysiological or neuroimaging measures. Uncontrolled studies and case reports were included only as lower-level, hypothesis-generating evidence. Broader TMS, non-DOC, animal, or preclinical mechanistic studies were used only to support plausible mechanisms and were interpreted as indirect evidence. Because this is a narrative review, no formal PRISMA-based screening, risk-of-bias scoring, or quantitative pooling was performed.

## 2 Overview of transcranial magnetic stimulation techniques

Consistent with this evidence-selection strategy, we distinguish therapeutic rTMS from diagnostic or probing TMS approaches. While TMS encompasses multiple modalities, repetitive TMS (rTMS) is the primary modality used for therapeutic intervention in DOC (Klomjai et al., 2015). In contrast, single-pulse and paired-pulse TMS are used in DOC mainly as neurophysiological probes to characterize corticospinal integrity and intracortical excitability/inhibition, and to support biomarker-oriented stratification rather than direct therapy (Ferreri et al., 2011). Accordingly, we use “therapeutic evidence” to refer to studies in which repeated or patterned stimulation is associated with clinically meaningful outcomes, such as CRS-R improvement, transition in diagnostic category, or sustained behavioral change. By contrast, “biomarker” or “probing” evidence refers to TMS-EEG, EEG, neuroimaging, MEP, or connectivity measures that characterize residual network capacity, excitability, or readiness for stimulation. These biomarker findings may support diagnosis, prognosis, or patient stratification, but they should not be interpreted as proof of therapeutic efficacy unless linked to clinical outcome improvement. This distinction is summarized in Table 1. The therapeutic rationale for rTMS, conversely, lies in its potential to induce lasting changes in cortical excitability and neuroplasticity (King and Tang, 2024). These effects are largely frequency dependent: high-frequency rTMS (HF-rTMS; ≥5 Hz) is thought to induce excitatory effects, whereas low-frequency (LF-rTMS; ≤1 Hz) is associated with inhibitory effects (Lefaucheur, 2019). Recently, patterned protocols such as theta-burst stimulation (TBS) have been developed to induce similar effects in much shorter timeframes. Intermittent TBS (iTBS) typically produces facilitatory effects, whereas continuous TBS (cTBS) is inhibitory (Huang et al., 2005). Deep TMS (e.g., H-coils) has established clinical use in some neuropsychiatric conditions; however, therapeutic evidence in DOC remains limited (Di Passa et al., 2024). While sTMS and ppTMS serve as essential diagnostic tools, rTMS, with its ability to induce durable changes in brain activity, forms the basis of therapeutic interventions for DOC, which will be the focus of this review.

**Table 1:** Distinguishing diagnostic/prognostic TMS-EEG roles from therapeutic rTMS roles in DOC.

Evidence type	Main role in DOC	Typical readouts	Interpretive boundary	Ref
Diagnostic/probing TMS-EEG or related biomarkers	Detect residual network capacity, covert responsiveness, or corticospinal integrity	TEPs, long-range propagation, EEG/fMRI/PET connectivity, MEPs	Supports diagnosis, prognosis, or stratification; not proof of therapeutic efficacy	Arai et al. (2021), Bai et al. (2024)
Therapeutic rTMS/iTBS evidence	Test whether repeated or patterned stimulation changes clinically meaningful outcomes	CRS-R improvement, diagnostic transition, sustained behavioral change	Requires clinical outcome evidence, preferably from sham-controlled or otherwise controlled studies	Bai et al. (2024), O’Neal et al. (2021)
Post-stimulation biomarker changes	Explore mechanisms, target engagement, and readiness for response	EEG/TMS-EEG/fMRI/PET changes after stimulation; connectivity or effective-information-flow changes	Intermediate mechanistic evidence only unless linked to clinically meaningful improvement	Bai et al., 2024

TMS-EEG and related biomarkers may identify residual capacity, covert responsiveness, or readiness for stimulation, whereas therapeutic rTMS evidence requires clinically meaningful outcome change. Post-stimulation biomarker changes may support mechanistic plausibility or target engagement, but these evidence types should not be treated as interchangeable.

## 3 Neurobiological mechanisms of rTMS in disorders of consciousness

**Figure 1:**
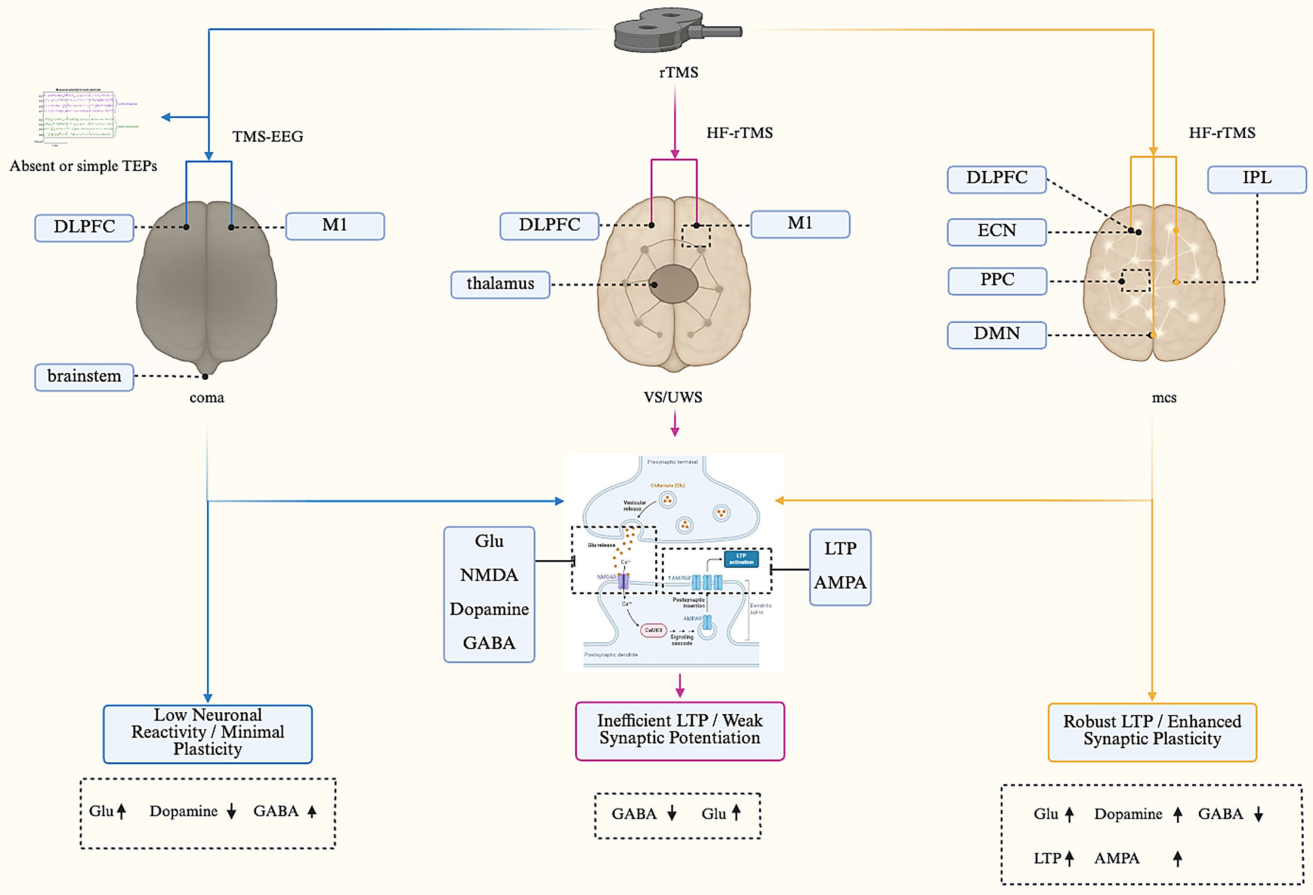
Simplified conceptual illustration of state-dependent rTMS hypotheses in disorders of consciousness.

The putative therapeutic rationale for rTMS in DOC is based on its capacity to modulate neural activity across multiple levels, from synaptic excitability to large-scale networks. However, most mechanistic evidence for LTP/LTD-like plasticity, glutamate/GABA modulation, dopamine release, BDNF-related neuroplasticity, and network reorganization comes from broader TMS literature, non-DOC human studies, or preclinical models. In DOC, these mechanisms should therefore be treated as plausible biological pathways that may help interpret clinical and biomarker observations, rather than as established subtype-specific causal mechanisms (Huang et al., 2023). The efficacy of these neuromodulatory effects is not uniform and is likely constrained by the patient’s underlying neurobiological state, which differs across the DOC spectrum (Wu et al., 2021). This section outlines core mechanisms that may contribute to rTMS effects; Figure 1 is used only as a simplified, hypothesis-generating illustration after these mechanistic uncertainties have been acknowledged.

### 3.1 Synaptic plasticity and metaplasticity: foundations and constraints of lasting change

The ability of rTMS to induce lasting therapeutic effects is thought to rely on its ability to modulate synaptic plasticity through mechanisms analogous to long-term potentiation (LTP) and long-term depression (LTD) (King and Tang, 2024). High-frequency rTMS (HF-rTMS) protocols, which are predominantly used in DOC, are believed to promote LTP. At the synaptic level, these effects are commonly discussed in terms of activity-dependent glutamatergic signaling and receptor trafficking that are consistent with LTP/LTD-like mechanisms; however, in DOC populations, direct molecular evidence is limited and much of this interpretation is inferred from non-DOC cohorts and preclinical models (Gersner et al., 2011; Sharbafshaaer et al., 2024). Conversely, low-frequency rTMS (LF-rTMS) can induce LTD, weakening synaptic connections, although this modality is less commonly used therapeutically in DOC (Lenz and Vlachos, 2016). Thus, the capacity to induce LTP is not an all-or-none phenomenon and may be important for therapeutic response. In this review, LTP/LTD-like terminology is therefore used as a mechanistic analogy rather than direct molecular proof in DOC. Residual plasticity may help indicate whether the injured brain is more likely to support fine-tuning or first requires readiness-oriented probing, but it should be considered a candidate stratification marker rather than an established treatment-selection rule.

In this context, neuroplasticity should be distinguished from metaplasticity. Neuroplasticity refers to activity-dependent changes in synaptic strength, excitability, and connectivity. Metaplasticity refers to the prior neural state that governs the threshold, direction, and durability of subsequent plastic changes. This distinction is clinically relevant because local TMS responses or LTP-like excitability changes do not necessarily translate into recovery of consciousness; meaningful improvement requires coordinated reconfiguration of distributed thalamo-cortical and frontoparietal networks. Accordingly, the “network rebooting versus network fine-tuning” terminology may be used cautiously to describe how residual plasticity, and metaplastic readiness might constrain network-level reorganization, without implying that distinct subtype-specific mechanisms have been established. These metaplastic conditions are dynamic across both the lifespan and the course of brain injury. Developmental periods, adolescence, and aging may differ in their capacity for adaptive plasticity, while severe brain injury may further alter responsiveness to stimulation through secondary injury cascades and later chronic remodeling. Thus, the same rTMS protocol may have different effects depending not only on DOC subtype, but also on developmental stage, injury stage, and the evolving metaplastic state of the injured brain (Karabanov et al., 2015; Masel and DeWitt, 2010; Müller-Dahlhaus and Ziemann, 2015; Sundman et al., 2020).

Therefore, the rebooting/fine-tuning distinction should be read as a conceptual extrapolation from current clinical and neurophysiological observations, rather than as direct evidence that distinct biological mechanisms have already been validated for each DOC subtype.

### 3.2 Neurotransmitter and neurotrophic modulation of plasticity

The induction of synaptic plasticity is tightly coupled with the modulation of the brain’s neurochemical milieu (Sharbafshaaer et al., 2024). One plausible, partly indirect mechanism is modulation of cortical excitation–inhibition balance, primarily discussed in relation to glutamate (Glu) and gamma-aminobutyric acid (GABA) systems. However, DOC-specific evidence directly measuring rTMS-induced glutamate/GABA changes remains limited, and this mechanism is largely inferred from broader TMS physiology studies (Du et al., 2018). In broader TMS literature, HF-rTMS is thought to shift cortical excitation–inhibition balance toward a plasticity-permissive state (Du et al., 2018). This shift creates a synaptic environment that is more conducive to LTP and may also influence metaplastic thresholds that determine whether subsequent stimulation produces facilitatory, inhibitory, or negligible effects (Karabanov et al., 2015).

Furthermore, evidence from non-DOC TMS studies suggests that prefrontal stimulation can influence dopaminergic pathways. Whether such dopaminergic modulation contributes to arousal or goal-directed behavior in DOC remains inferential, although it provides a plausible mechanistic link for future study (Cho and Strafella, 2009). Preclinical and broader neuromodulation studies also suggest that repeated stimulation can influence neurotrophic pathways, including brain-derived neurotrophic factor (BDNF)-related plasticity. Because BDNF supports neuronal survival, growth, and synaptic remodeling, it may provide a plausible molecular pathway through which repeated stimulation could support longer-term plasticity, although direct DOC-specific evidence remains limited (Gersner et al., 2011). Collectively, these actions suggest that rTMS may influence a neurochemical and neurotrophic milieu that is permissive for plasticity-related change.

### 3.3 Network-level reorganization: from local stimulation to global effects

These cellular-level changes may manifest as the modulation of large-scale brain networks, but this translation is neither automatic nor linear. In DOC, local synaptic or corticospinal responsiveness should therefore be distinguished from network-level expression across complex thalamo-cortical and frontoparietal interconnections. Rather than a purely focal intervention, rTMS can be conceptualized as a perturbation of a specific node within a complex, interconnected system, thereby influencing the activity and connectivity of functionally related, yet anatomically distant, brain regions (Beynel et al., 2020; Naro et al., 2017).

In the context of DOC, consciousness is believed to emerge from the coordinated activity of specific brain networks, most notably the default mode network (DMN), the executive control network (ECN), and the salience network (Li et al., 2023). Severe brain injury disrupts the integrity and communication within and between these networks. Therapeutic rTMS is hypothesized to modulate this dysfunctional connectivity; however, connectivity changes should be treated as intermediate biomarker readouts unless they are accompanied by clinically meaningful behavioral outcomes (Xia et al., 2019). For example, stimulation of a key hub such as the dorsolateral prefrontal cortex (DLPFC) or the posterior parietal cortex (PPC) has been reported to alter functional connectivity within the DMN and communication with subcortical structures such as the thalamus, which is critical for maintaining arousal and cortical activity (Liu et al., 2018; Xia et al., 2019). In practice, these network-level readouts provide measurable intermediate phenotypes for DOC rather than standalone therapeutic endpoints. Within the heuristic, what is termed “rebooting” would be expected to depend mainly on baseline reactivity/propagation, whereas “fine-tuning” would depend more on residual frontoparietal integration and capacity for connectivity reconfiguration; however, this distinction remains an interpretive hypothesis rather than a validated subtype-specific rule. Ultimately, the journey from a local magnetic pulse to a clinical outcome is a multistage process that is critically dependent on this preexisting neurobiological landscape. This landscape includes not only the current level of consciousness but also age-related network maturation or degeneration, etiology-specific patterns of structural disconnection, injury chronicity, and systemic or environmental modifiers of cortical excitability, plasticity, and metaplastic readiness. These issues are therefore considered as patient-level modifiers in the clinical sections below.

This figure provides a simplified conceptual illustration of state-dependent rTMS hypotheses across coma, VS/UWS, and MCS. It is intended as a conceptual heuristic for organizing current evidence and hypotheses, not as a validated physiological model or a clinical treatment algorithm. Biomarker features shown in the figure should be interpreted as readiness or probing markers unless they are linked to clinical outcome changes in therapeutic studies. (A) Coma (left): TMS-evoked potentials (TEPs) are typically absent or simple, reflecting limited cortical reactivity and reduced capacity for long-range propagation. (B) VS/UWS (middle): High-frequency rTMS (HF-rTMS) is applied to candidate cortical targets to engage residual thalamo-cortical/frontoparietal circuits; however, plasticity-related mechanisms are hypothesized to be constrained, consistent with weak network propagation and heterogeneous behavioral responses. (C) MCS (right): More preserved large-scale network connectivity (e.g., ECN/DMN) may provide a more plausible substrate for HF-rTMS effects at key network nodes (e.g., DLPFC/IPL), and responders may show network-level and plasticity−/metaplasticity-permissive changes. rTMS, repetitive transcranial magnetic stimulation; VS, vegetative state; UWS, unresponsive wakefulness syndrome; MCS, minimally conscious state; TEPs, TMS-evoked potentials; DMN, default mode network; ECN, executive control network; DLPFC, dorsolateral prefrontal cortex; M1: primary motor cortex; HF-rTMS, high-frequency rTMS; PPC, posterior parietal cortex; IPL, inferior parietal lobule; Glu, glutamate; GABA, gamma-aminobutyric acid; LTP, long-term potentiation; CaMKII, calcium/calmodulin-dependent kinase II; NMDA, N-methyl-D-aspartate; AMPA, *α*-amino-3-hydroxy-5-methyl-4-isoxazolepropionic acid (AMPA) receptors.

## 4 State-dependent modulation across DOC subtypes

The evidence synthesis in this section is organized first around the strength and heterogeneity of available clinical and biomarker evidence. The framework proposed here should therefore be understood as a hypothesis-generating conceptual heuristic rather than a validated treatment algorithm. Direct evidence supports a broad response gradient, with relatively more consistent CRS-R improvement in MCS, heterogeneous findings in VS/UWS, and insufficient therapeutic evidence in coma. However, this gradient may be distorted by baseline diagnostic uncertainty, fluctuating arousal, and unrecognized cognitive motor dissociation, particularly in patients behaviorally classified as VS/UWS. Neurophysiological findings further suggest that residual cortical reactivity, long-range propagation, and frontoparietal connectivity differ across DOC subtypes. These neurophysiological findings are useful for stratification and readiness assessment, but they do not by themselves demonstrate that rTMS produces clinically meaningful recovery. The terms “network rebooting” and “network fine-tuning” are therefore used to organize these clinical and biomarker observations into testable hypotheses, not to prescribe specific targets, frequencies, or treatment strategies for individual subtypes. This qualification is especially important for coma, for which the current literature supports TMS-EEG-based assessment of residual reactivity more strongly than therapeutic rTMS guidance.

In VS/UWS, the central therapeutic challenge is not to modulate complex cognition but to overcome profound neuronal inertia and limited network responsiveness. In coma, by contrast, the first challenge is to determine whether sufficient residual cortical reactivity exists to justify therapeutic stimulation trials. Within this cautious interpretation, “network rebooting” refers to the hypothesis that stimulation of a putative critical network node might help re-establish basic thalamo-cortical reactivity and distributed responsiveness in selected patients with sufficient residual reactivity. Consistent with this view, TMS-EEG studies in these states often show absent, markedly attenuated, or predominantly local TMS-evoked potentials (TEPs), which may indicate constrained capacity for long-range causal interactions rather than subtle network modulation (Ragazzoni et al., 2013; Sarasso et al., 2014). By contrast, in MCS the primary research question is less whether stimulation can engage the system and more whether partially preserved frontoparietal networks can be modulated in a clinically meaningful direction. In this sense, “network fine-tuning” is a hypothesis about how future biomarker-guided studies might test stimulation of patient-specific dysfunctional nodes and connections.

To avoid implying a clinical algorithm, Table 2 provides a descriptive map of how the rebooting/fine-tuning heuristic relates to currently reported study features, including conceptual questions, reported targets, reported target–parameter combinations, evidence status, and candidate readiness/probing markers. Importantly, the targets and parameter ranges in Table 2 are restricted to those reported in the studies summarized in Table 3 and are intended as a transparent mapping of existing practice patterns, including both mechanistically motivated and pragmatically selected targets, rather than prescriptive recommendations. Table 2 should therefore be read as a conceptual heuristic for hypothesis generation and study design, not as a treatment algorithm, clinical decision rule, or evidence-based parameter-selection guide. No target, frequency, intensity, dose, or biomarker listed in the table should be interpreted as sufficient for clinical decision-making outside a study protocol.

**Table 2:** Descriptive conceptual map of the “network rebooting vs. network fine-tuning” heuristic across DOC subtypes (reported study features only; not a treatment algorithm).

DOC subtype	Conceptual research question	Targets reported in Table 3 (descriptive only)	Reported target–parameter combinations from Table 3 (examples; not comparable across studies)	Evidence strength represented in Table 3	Readiness/probing biomarkers (not therapeutic outcomes; conceptual examples)
Coma	Research question: whether residual reactivity can justify exploratory stimulation studies	Not established (evidence gap in Table 3)	Not established in Table 3	Evidence gap	Primarily relevant for future study eligibility rather than current therapeutic selection: residual cortical reactivity (EEG/TMS-EEG), preserved long-range propagation, and/or partially preserved connectivity on fMRI; age, etiology, acute injury stage, sedation, metabolic suppression, systemic instability, access to neurocritical care, resource setting, and severely altered metaplastic readiness may confound assessment.
VS/UWS	Research hypothesis: whether residual networks can be engaged beyond local reactivity	M1; DLPFC; PPC	rTMS: 20 Hz 90% RMT (M1); 10 Hz 100% RMT (rDLPFC); 5 Hz 80% RMT (lDLPFC); 10 Hz 90% RMT (lPPC). iTBS: 50 Hz triplets at 5 Hz, 600 pulses, 80% RMT (lDLPFC).	Sham-controlled crossover RCT/sham-controlled RCT/controlled studies	Repeated CRS-R confirmation and arousal assessment; EEG reactivity; TMS-EEG responses beyond local cortex; evidence of covert command-following or cognitive motor dissociation when available; partially preserved frontoparietal/thalamocortical connectivity; connectivity increases after stimulation should be interpreted as intermediate biomarkers rather than standalone therapeutic endpoints. Interpretation should account for age, etiology, injury chronicity, spontaneous recovery trajectory, access to rehabilitation resources, resource setting, and metaplastic thresholds for network re-engagement.
MCS	Research hypothesis: whether preserved networks can be modulated toward clinically meaningful improvement	M1; DLPFC; IPL (P3/P4)	M1: 20 Hz; 90% RMT; DLPFC: 10 Hz; 90% RMT; 1,000 pulses/day × 20 days; Bilateral IPL (P3/P4) + tDCS: rTMS 5 Hz; 70% RMT + anodal tDCS 1.5 mA (case-report combination protocol only; not separable as rTMS-specific evidence)	Case reports/small clinical studies	Preserved behavioral signs (MCS−/MCS+); more complex TMS-EEG responses; preserved resting-state connectivity; measurable EEG/fMRI connectivity change following stimulation; age, etiology, developmental or degenerative context, resource setting, and access to repeated assessment and rehabilitation may influence the available plasticity reserve and metaplastic readiness for fine-tuning.

This table is intended only to organize reported study features and hypotheses. It should not be used to select stimulation targets, parameters, or patients in clinical practice. Biomarkers listed here are candidate research stratification variables, not validated therapeutic eligibility criteria. DOC, disorders of consciousness; VS/UWS, vegetative state/unresponsive wakefulness syndrome; MCS, minimally conscious state; rTMS, repetitive transcranial magnetic stimulation; iTBS, intermittent theta-burst stimulation; DLPFC, dorsolateral prefrontal cortex; PPC, posterior parietal cortex; IPL, inferior parietal lobule; M1, primary motor cortex; RMT, resting motor threshold; CRS-R, Coma Recovery Scale–Revised; EEG, electroencephalography; fMRI, functional magnetic resonance imaging; tDCS, transcranial direct current stimulation.

**Table 3:** Summary of key studies on rTMS for DOC, including available population and etiology-related information.

DOC subtype	TMS mode	Study design	Sample size	Population and etiology	Generalizability considerations	Therapeutic targets	Therapeutic parameters	Main findings	Ref
VS	rTMS	Randomized, sham-controlled trial (crossover design)	*n* = 11	Adult/chronic VS; 9 post-anoxic and 2 post-traumatic patients; 9–85 months after injury.	Small sample; chronic VS population; predominantly post-anoxic etiology; M1 stimulation only; pediatric applicability unclear.	Left M1	20 Hz; 90% RMT	No significant difference in CRS-R scores between the rTMS and sham stimulation periods (crossover design).	Cincotta et al., 2015
VS	rTMS	Controlled clinical study (retrospective cohort; no sham/randomization)	*n* = 32 (15 rTMS, 17 control)	Adults aged 18–75 years; VS caused by brain injury; early DOC/VS population treated after admission.	Single-center retrospective cohort; non-randomized allocation; no sham stimulation; specific etiological distribution not fully stratified.	Right DLPFC	10 Hz; 100% RMT	The rTMS group showed significantly greater improvement in CRS-R scores compared to the control group. 86.7% of patients in the rTMS group transitioned from VS to MCS. MEP latency and CMCT were also significantly reduced.	Ge et al., 2021
VS/PVS	rTMS	Prospective, open-label randomized controlled trial (false/sham stimulation as control)	*n* = 48 (24 rTMS, 24 sham)	Adults aged 18–80 years; persistent vegetative state lasting >3 months; etiology not stratified in the main table.	Single-center open-label randomized study; false/sham stimulation used as control; limited reporting of etiology and long-term follow-up.	Left DLPFC	5 Hz; 80% RMT	The rTMS group showed significantly greater improvement in CRS-R scores and shorter brainstem auditory evoked potential (BAEP) latencies.	Zhang et al., 2021
VS	iTBS	Sham-controlled, randomized crossover study (single-session iTBS)	*n* = 18	VS patients; single-session iTBS study; detailed age and etiological distribution should be interpreted according to the original cohort description.	Single-session physiological study; short-term EEG-based outcomes; behavioral efficacy and long-term therapeutic effects remain uncertain.	Left DLPFC	50 Hz triplet bursts at 5 Hz; 600 pulses; 80% RMT	A single session of iTBS induced a significant increase in EEG delta power in the prefrontal region compared to sham.	Huang et al., 2024
VS/UWS	rTMS	Crossover, randomized, double-blind, sham-controlled study	*n* = 20	Adult VS/UWS patients with DoC; crossover randomized double-blind sham-controlled study; etiological heterogeneity should be considered.	Small sample; short treatment and washout periods; response may vary by etiology, especially hypoxic–ischemic injury.	Left Posterior Parietal Cortex (PPC)	10 Hz; 90% RMT	Significant improvement in CRS-R scores in the rTMS group. TMS-EEG showed increased connectivity, particularly in responders.	Xu et al., 2023
MCS	rTMS	Case report	*n* = 1	Single MCS case; age and etiology should be interpreted as case-specific.	Case report; no control condition; findings are hypothesis-generating and cannot be generalized.	Left M1	20 Hz; 90% RMT	CRS-R score increasing from 14 to 19; Increased power in the α, *β*, and δ frequency bands of electroencephalograms (EEGs).	Piccione et al., 2011
MCS, VS/UWS	rTMS	Prospective single-arm study (uncontrolled) with blinded outcome assessment	*n* = 16 (5 MCS, 11 VS/UWS)	Adults with chronic DOC; 5 MCS and 11 VS/UWS; age range 23–67 years; DOC duration >3 months.	Prospective single-arm study without sham control; small sample; etiologies not analyzed as subgroups; natural recovery cannot be fully excluded.	Left DLPFC	10 Hz; 90% RMT; 1,000 pulses/day × 20 days	Superior improvement in MCS patients, with significant CRS-R gains (5/5 improved), versus nonsignificant gains in the VS/UWS group (4/11).	Xia et al. 2017
MCS	rTMS + tDCS	Case report (with 1 comparator/control patient; combined NIBS)	*n* = 1 (+ 1 control)	Single MCS patient with brainstem hemorrhage 1 month prior; one comparator/control patient receiving routine treatment.	Case report with one comparator; combined tDCS+rTMS protocol; target selection was individualized by EEG/fMRI, limiting generalizability.	Bilateral inferior parietal lobes (IPLs; P3 & P4)	rTMS: 5 Hz; 70% RMT; tDCS: 1.5 mA anodal.	The patient’s CRS-R score improved from 10 to 19 over 1 month, with associated increases in EEG/fMRI connectivity markers. The control patient showed minimal change.	Lin et al., 2019

This table summarizes key therapeutic rTMS/iTBS studies selected for narrative synthesis rather than a PRISMA-based systematic evidence map. Study design is shown to indicate evidence strength; findings should be interpreted cautiously in light of sample size, population characteristics, DOC subtype, follow-up, resource setting, and access-related factors. Cross-subtype and cross-target comparisons remain provisional because positive and negative findings come from heterogeneous and not directly comparable study designs, small samples, variable outcome definitions, and different target–parameter combinations. DOC, disorders of consciousness; TMS, transcranial magnetic stimulation; rTMS, repetitive TMS; VS, vegetative state; MCS, minimally conscious state; PVS, persistent vegetative state; UWS, unresponsive wakefulness syndrome; DLPFC, dorsolateral prefrontal cortex; lDLPFC, left dorsolateral prefrontal cortex; rDLPFC, right dorsolateral prefrontal cortex; PPC, posterior parietal cortex; RMT, resting motor threshold; GCS, Glasgow Coma Scale; CRS-R, Coma Recovery Scale–Revised; EEG, electroencephalogram; BAEP, brainstem auditory evoked potential; MEP, motor evoked potential; CMCT, central motor conduction time; M1, primary motor cortex; CGI-I, Clinical Global Impression-Improvement; iTBS, intermittent theta-burst stimulation; tDCS, transcranial direct current stimulation; DMN, default mode network; ECN, executive control network; fMRI, functional magnetic resonance imaging.

Target selection in this literature should also be interpreted descriptively rather than prescriptively, because current choices appear to reflect a mixture of mechanistic rationale, historical convention, anatomical accessibility, and methodological convenience. The DLPFC has a partial mechanistic rationale because of its role in executive/frontoparietal networks and its putative influence on thalamo-cortical and default-mode/executive-control interactions, but its frequent use also reflects scalp accessibility, feasibility of repeated stimulation, and accumulated precedent in noninvasive neuromodulation studies (Huang et al., 2023; Wan et al., 2024; Xia et al., 2017). M1 appears to be driven more strongly by historical and methodological considerations than by a DOC-specific awareness-network rationale, because it is anatomically and physiologically accessible, allows motor-threshold calibration and MEP-based probing, and was inherited from early stimulation protocols (Huang et al., 2023; Yang et al., 2023). By contrast, PPC and IPL targets are more explicitly mechanism- and network-oriented, reflecting their involvement in posterior cortical integration, frontoparietal connectivity, and awareness-related networks; however, their use is still constrained by feasibility, coil positioning, neuronavigation availability, and small-sample evidence (Lin et al., 2019; Xu et al., 2023). Thus, current target selection should be understood as a pragmatic and partly theory-informed practice pattern rather than a set of validated subtype-specific rules. Differences across DLPFC, M1, PPC, and IPL studies should therefore not be interpreted as direct evidence that one target is superior for a given DOC subtype (Barra et al., 2022; Yang et al., 2023).

Given the heterogeneous study designs and small sample sizes in this field, we interpret clinical effects with explicit attention to evidence strength (e.g., sham-controlled trials vs. uncontrolled or non-randomized studies) and treat mechanistic implications as hypothesis-generating when direct DOC evidence is limited. Future non-randomized interventional studies should therefore prespecify causal assumptions and distinguish prognostic associations from treatment effects.

Consistent with the heterogeneous evidence summarized above and cautiously illustrated in Figure 1, the patient’s position along the DOC spectrum may influence hypotheses about therapeutic engagement, but it should not be used to determine treatment selection on its own. The profound neuronal inertia in coma presents a formidable barrier to neuromodulation. In VS/UWS, while a basic substrate for stimulation exists, inefficient plasticity often limits significant clinical gains (Chen et al., 2018; Cincotta et al., 2015). In contrast, the more preserved residual network readiness in MCS may provide a more plausible substrate for rTMS-induced modulation, although this inference remains provisional and requires confirmation in larger controlled studies (O'Neal et al., 2021).

The apparent therapeutic effect of rTMS appears to vary across DOC subtypes and may be influenced by the degree of preserved neurological function. A growing body of research has investigated rTMS interventions in patients ranging from coma to VS/UWS and MCS, suggesting a possible hierarchy of clinical responsiveness, with patients in higher states of consciousness often showing greater gains. However, pooled interpretive conclusions remain provisional because study designs, sample sizes, control conditions, stimulation protocols, and outcome definitions vary substantially across subtypes (Table 3). The following sections synthesize evidence across DOC subtypes, with attention to clinical findings, mechanistic rationale, and study limitations. Table 3 supports this synthesis by summarizing key therapeutic rTMS/iTBS studies identified through the narrative evidence-selection process, including study design, population characteristics, stimulation parameters, main findings, and generalizability limitations.

### 4.1 Coma: evidence gap and probing for residual reactivity

Compared with VS/UWS and MCS, coma remains the least established context for therapeutic rTMS. Accordingly, this section is intended primarily to outline residual-reactivity assessment and future study requirements rather than to provide evidence-based therapeutic guidance (Chen et al., 2022). The profound cerebral dysfunction in coma distinguishes it from VS/UWS or MCS and makes both responsiveness and safety interpretation especially uncertain (Porcaro et al., 2021). While TMS-EEG can assess cortical integrity, findings in coma typically reveal severe network impairment, characterized by absent or markedly attenuated TMS-evoked potentials (TEPs) (Chung et al., 2015; Gosseries et al., 2014; Rosanova et al., 2012). Accordingly, translating diagnostic observations into reliable therapeutic effects in coma is challenging. In addition, network-level effects reported in less severe DOC (e.g., changes consistent with improved thalamo-cortical coupling after HF-rTMS) may be difficult to elicit in a profoundly suppressed system (Bai et al., 2016; Xia et al., 2019).

Importantly, coma is clinically heterogeneous, and the acute neurocritical care setting differs substantially from prolonged DOC trajectories. Etiology-specific mechanisms, such as traumatic diffuse axonal injury, hypoxic–ischemic injury, vascular injury, infection, toxic-metabolic encephalopathy, or systemic inflammatory states, may produce different patterns of cortical excitability, thalamo-cortical disconnection, and recovery potential. Age and developmental stage further complicate interpretation, because pediatric coma and adult coma differ in baseline neurodevelopmental status, assessment tools, spontaneous recovery trajectories, and vulnerability to secondary injury (Duan et al., 2024; Pundole and Crawford, 2018). In acute coma, sedation/analgesia, metabolic derangements, and temperature management can transiently alter cortical excitability and EEG/TMS readouts, complicating patient stratification and interpretation of “responsiveness” (Edlow et al., 2021). Therefore, future exploratory rTMS studies in coma, if undertaken, should explicitly account for these confounders and adopt a readiness-oriented approach (e.g., EEG reactivity or TMS-EEG responses beyond the local cortex) when feasible. In summary, while TMS-EEG has clear value for characterizing coma pathophysiology and residual cortical responsiveness, the therapeutic role of rTMS—especially in the acute phase—remains uncertain. The coma-related “rebooting” concept should therefore be interpreted as a future research hypothesis rather than evidence-based therapeutic guidance. Rigorous, adequately powered studies designed specifically for coma are needed to determine whether any subgroup benefits and to define feasible targets, timing, dosing strategies, and safety requirements.

### 4.2 VS/UWS: challenges in neuromodulation

Based on the heterogeneous evidence summarized above, VS/UWS can be cautiously described as closer to a “network rebooting” research hypothesis, where stimulation would need to increase readiness for distributed network responses.

Reviews suggest that the therapeutic benefit is often modest in the VS/UWS population compared with the MCS population (Porcaro et al., 2021; Ragazzoni et al., 2013). In this section, we focus on rTMS evidence aimed at promoting recovery in VS/UWS.

Several trials have reported positive outcomes. For example, HF-rTMS over the DLPFC has been shown to improve CRS-R scores and favorably modulate EEG indices in patients with VS/UWS (Zhang et al., 2021). Similarly, another study reported that rTMS improved electrophysiological markers such as BAEPs and MEPs, which correlated with a high rate of transition to MCS (Ge et al., 2021). In interpreting these studies, CRS-R changes should be considered clinical therapeutic outcomes, whereas EEG, BAEP, MEP, or connectivity changes should be treated as biomarker or probing evidence that may support mechanistic interpretation but does not by itself establish behavioral efficacy.

However, the therapeutic window for patients with VS/UWS appears narrower than that for patients with other DOC subtypes. Meta-analytic evidence indicates that superior outcomes are often observed in patients with MCS, specific etiologies such as stroke, or those who receive intervention earlier (<3 months postinjury) (O'Neal et al., 2021). This suggests that positive pooled estimates across all DOCs should not be directly extrapolated to VS/UWS, where the specific benefit may be more modest and more dependent on target, timing, etiology, and baseline network integrity (Zheng et al., 2023). In addition, apparent treatment responses in behaviorally diagnosed VS/UWS may partly reflect baseline misclassification or unrecognized covert consciousness. Patients with covert motor dissociation, fluctuating arousal, or subtle but missed behavioral signs may be classified as VS/UWS at enrollment yet have a neurophysiological profile closer to MCS, which could inflate apparent subtype-specific responsiveness if not systematically assessed. This interpretation is further complicated by population heterogeneity: patients with traumatic, anoxic, vascular, infectious, or toxic-metabolic etiologies may differ in residual structural connectivity and spontaneous recovery potential, while pediatric and adult VS/UWS populations may not share the same natural history or neuroplastic capacity. A notable sham-controlled study by Cincotta et al. (2015), for example, reported no significant clinical improvement after 20 Hz rTMS to the M1 in patients with VS/UWS. Such negative sham-controlled findings are informative because they suggest that residual cortical excitability or stimulation feasibility alone is insufficient to guarantee behavioral improvement, and that target selection, etiology, baseline network integrity, and outcome timing may critically shape response. Because VS/UWS studies have used different cortical targets, including M1, DLPFC, and PPC, together with different frequencies, intensities, and dosing schedules, discrepant findings cannot be attributed solely to DOC subtype. The neurobiological basis for this limited efficacy likely lies in the profound neurological impairment characteristic of VS/UWS. Indeed, TMS-EEG studies suggest that cortical responsiveness and large-scale information integration are often severely compromised in these patients, providing a limited substrate for effective neuromodulation compared with those in milder states of consciousness (Sinitsyn et al., 2020). Therefore, rigorous RCTs are essential to verify efficacy and optimize protocols that address the unique challenges of VS/UWS.

### 4.3 MCS: a provisional window for rTMS response

Based on available but unequal evidence, MCS is cautiously discussed as most closely aligned with a “network fine-tuning” hypothesis, in which stimulation may be more likely to modulate partially preserved frontoparietal integration. This does not mean that the MCS evidence base is methodologically stronger than that for VS/UWS; rather, the observed clinical signal appears more consistent despite reliance on small samples, mixed-subtype cohorts, and partly uncontrolled evidence. Nevertheless, MCS should not be regarded as a homogeneous state. Age, etiology, time since injury, and the distinction between MCS − and MCS + may all influence whether residual networks are sufficiently preserved to support rTMS-induced modulation.

Compared with patients in coma or VS/UWS, patients in an MCS retain a greater degree of residual neural function, making them a more plausible population for both advanced diagnostics and therapeutic rTMS testing (Faugeras et al., 2018). Diagnostically, TMS-based techniques are crucial for characterizing this residual function. For example, single-pulse TMS can assess corticospinal tract integrity via motor-evoked potentials (MEPs), whereas TMS-EEG reveals the state of cortical excitability and network connectivity (Bai et al., 2024; Zhao et al., 2024). Studies generally show that TMS-evoked potentials (TEPs) in MCS patients have distinct spatiotemporal signatures compared with those in VS/UWS patients, reflecting more preserved cortical information processing (Ragazzoni et al., 2013). Specifically, the retention of long-range functional connectivity is more characteristic of MCS, suggesting a greater capacity for cortical integration and a plausible substrate for therapeutic testing; however, these diagnostic/prognostic markers do not themselves prove rTMS efficacy (Kotchoubey et al., 2013; Liu et al., 2018).

Recently, patients with MCS have been divided into two subtypes, namely, MCS − (patients exhibiting only nonreflexive behaviors such as visual pursuit) and MCS + (patients capable of command-following), the differences between which are key factors influencing the efficacy of rTMS (Bruno et al., 2012). A key study by Xia et al. investigating 10 Hz rTMS on the DLPFC in 16 patients with chronic DOC (5 MCS and 11 VS/UWS) reported encouraging but differential outcomes. While a total of nine patients (all 5 patients with MCS and 4 of 11 patients with VS/UWS) presented increases in CRS-R scores, the therapeutic effects were statistically significant and more pronounced in the MCS cohort. On the basis of clinical impression scores, several patients were rated as having ‘improved’ or ‘considerably improved’, suggesting, within the limitations of a small mixed-subtype cohort, that MCS may represent a more responsive substrate for therapeutic rTMS testing (Xia et al., 2017). A major meta-analysis assessing covert consciousness revealed that patients with MCS were significantly more likely than those with VS/UWS to demonstrate command-following via brain activity (32% vs. 14%) and to show preserved cortical functional connectivity in response to external stimuli (55% vs. 26%) (Kondziella et al., 2016). This suggests that MCS patients, as a group, may have a more responsive neural architecture. These observations support the hypothesis that preserved command processing and network communication may make some MCS patients more amenable to rTMS-induced plastic or metaplastic modulation; however, this remains a mechanistic interpretation that requires prospective confirmation with clinical outcomes (Li et al., 2024; Xia et al., 2019; Xu et al., 2023).

Despite these findings, significant challenges remain in optimizing rTMS for MCS. Key issues include the individualization of stimulation parameters (e.g., frequency, intensity, and target selection on the basis of patient-specific network deficits) and the need to confirm the long-term sustainability of therapeutic effects (Fan et al., 2022). Future progress will require rigorous, adequately powered randomized controlled trials that incorporate multimodal biomarkers, including neuroimaging and electrophysiology, to test whether the more favorable signal observed in MCS can be replicated and translated into reproducible clinical benefit (Barra et al., 2022; Vitello et al., 2023).

## 5 Factors influencing therapeutic efficacy

The therapeutic response to rTMS in patients with DOC is highly variable, depending on a range of patient- and stimulation-specific factors (Chen et al., 2022; He et al., 2020; Pell et al., 2011). Patient-specific characteristics are critical determinants of outcomes. Etiology is one of the most important modifiers: patients with traumatic brain injury often show greater recovery potential than those with anoxic or hypoxic–ischemic injuries, whereas vascular, infectious, toxic-metabolic, and mixed etiologies may differ in lesion distribution, residual connectivity, systemic vulnerability, and spontaneous recovery trajectory (Smania et al., 2013; Whyte et al., 2009). Age at injury is another key modifier. Pediatric DOC should not be interpreted simply as a smaller version of adult DOC, because developmental stage influences behavioral assessment, cortical excitability, myelination, network maturation, and adaptive or maladaptive plasticity. In contrast, older adults may have reduced physiological reserve, more comorbidities, and greater pre-existing neurodegenerative or vascular burden. These lifespan-related differences may alter TMS-EEG readouts and rTMS responsiveness by shaping both neuroplastic capacity and the metaplastic state of the injured brain (Duan et al., 2024; Kunikullaya, 2025; Sundman et al., 2020).

Diagnostic classification itself is a major interpretive factor. Apparent rTMS responsiveness may be influenced by baseline misclassification, hidden cognitive motor dissociation, covert consciousness, or fluctuations in arousal and attention during behavioral assessment. Repeated CRS-R assessments, documentation of arousal state and medication/sedation exposure, and complementary EEG, TMS-EEG, fMRI, or PET markers may reduce classification error and improve biomarker-guided stratification. Without such procedures, apparent subtype-dependent effects may partly reflect differences in diagnostic sensitivity rather than true differences in neuromodulatory responsiveness.

Similarly, the baseline degree of residual network integrity, often assessed by EEG/TMS-EEG or related neurophysiological measures, is a candidate stratification marker for therapeutic response, but its predictive value requires prospective validation against clinically meaningful outcomes (He et al., 2020; Xu et al., 2024). The selection of parameters such as target, frequency, intensity, and stimulation duration is also important, because their interaction with the patient’s neurophysiological state may shape clinical outcomes (Guerra et al., 2014; Liu et al., 2023; Thibaut et al., 2019; Zheng et al., 2023). Therefore, future trials testing therapeutic efficacy should incorporate precise DOC subtype classification, explicitly justify whether target selection is mechanism-driven or primarily pragmatic, and prospectively evaluate individualized target–parameter adjustment strategies. Combination approaches, including rTMS paired with pharmacological agents such as amantadine or with other neuromodulation techniques, should be regarded as exploratory rather than clinically established. Current evidence is too limited and heterogeneous to support rational personalization of such combinations, and future studies should first clarify safety, timing, interaction effects, and whether any added benefit exceeds that of each component alone (Liu et al., 2025; Weaver et al., 2023). Future rTMS studies should therefore report, at minimum, age or developmental stage, sex, gender identity when appropriate and ethically collected, race and ethnicity when relevant to the study context, socioeconomic resources, geographic distance from specialized care, country or region of recruitment, resource setting, etiology, time since injury, acute versus chronic status, baseline CRS-R subdomain profile, sedation and medication exposure, comorbidities, neuroimaging-defined lesion burden, and the availability of rehabilitation resources. These variables are essential for distinguishing true neuromodulatory effects from spontaneous recovery, selection bias, context-dependent differences in clinical implementation, and variability in plastic or metaplastic responsiveness. They may also influence whether neuroprotective and neuromodulatory interventions are introduced proactively during acute care or reactively during chronic rehabilitation, thereby affecting treatment timing, adherence, follow-up completeness, and external validity. Standardized reporting of these variables would also make future datasets more suitable for AI-assisted and machine-learning-based prediction models and for causal-inference analyses that examine whether rTMS effects vary across etiological, developmental, intersectoral, and resource-related strata.

### 5.1 Equity, access, and resource-sensitive implementation

Beyond biological and stimulation-related factors, intersectoral, intersectional, and global implementation determinants may influence how rTMS evidence is interpreted and applied in DOC. Sex, gender identity, race, ethnicity, socioeconomic resources, insurance or reimbursement structures, caregiver availability, and geographic distance from specialized neurocritical care and neurorehabilitation centers may affect the likelihood of receiving early neuroprotective management, advanced diagnostic evaluation with TMS-EEG or neuroimaging, repeated rTMS sessions, and longitudinal follow-up (Fornari et al., 2025; Mollayeva et al., 2021). At the global level, resource-constrained communities and nations may face limited access to neurocritical care, EEG/TMS-EEG, MRI/fMRI, trained personnel, and repeated rehabilitation visits (GBD 2021 Nervous System Disorders Collaborators, 2024; Fornari et al., 2025), which can alter baseline patient characteristics, treatment timing, outcome ascertainment, and the apparent effectiveness of TMS/rTMS interventions. Moreover, colonial or extractive patterns in global health research may arise when data, analytic expertise, infrastructure, or patient populations from the Global South contribute to methods, devices, or publications that primarily benefit institutions and patients in the Global North (Khan et al., 2021; Lawrence and Hirsch, 2020). These factors are not established biological determinants of rTMS efficacy, but they may shape study enrollment, implementation feasibility, data interpretation, and generalizability. Therefore, future studies should transparently report resource setting and access-related variables, promote equitable data governance and authorship, and evaluate whether biomarker-informed neuromodulation can be implemented in ways that produce local benefit for participating communities. In this context, AI-assisted tools may help identify scalable, resource-sensitive triage pathways, but only if models are developed and validated using representative datasets and are monitored for algorithmic bias. Complementary causal diagrams, such as directed acyclic graphs, may help make explicit how intersectoral factors, resource setting, treatment timing, and baseline neurological status shape both access to rTMS and observed outcomes (Greenland et al., 1999).

## 6 Safety considerations

### 6.1 General risks and state−/setting-specific factors

Although rTMS has a generally favorable safety profile, applying it in DOC populations requires vigilance due to impaired neural networks and common polypharmacy. Safety considerations do differ meaningfully across coma, VS/UWS, and MCS, although these differences relate less to a simple hierarchy of intrinsic risk than to clinical setting, adverse-event detectability, monitoring feasibility, and confounding by sedation, systemic instability, or behavioral fluctuation (Huang et al., 2023; Kletzel et al., 2020). Because both responsiveness and risk interpretation are state-dependent, safety monitoring in DOC should not be treated as uniform across coma, VS/UWS, and MCS. Importantly, safety considerations are contingent upon the clinical environment. In neurocritical care settings, variables such as deep sedation, metabolic instability, thermoregulatory interventions, acute etiology, age, and developmental stage can modulate cortical excitability, thereby complicating risk stratification and response assessment. In chronic phases, primary concerns pivot toward physiological fragility and the long-term neuroplastic and metaplastic effects of sustained stimulation. Consequently, safety protocols must be tailored to the patient’s clinical course—differentiating between acute and chronic management stages—and should also account for implementation-related factors such as caregiver support, travel burden, access to EEG monitoring, continuity of follow-up, and the feasibility of safe monitoring in resource-constrained settings.

### 6.2 State-dependent safety monitoring across DOC subtypes

Safety monitoring should therefore be adapted to DOC subtype and clinical setting. The main difference is not necessarily a simple subtype-based hierarchy of intrinsic risk, but rather the way in which adverse events can be detected, monitored, and interpreted across coma, VS/UWS, and MCS. In coma, especially in acute neurocritical care, therapeutic rTMS remains exploratory rather than a routine therapeutic intervention. Safety interpretation is particularly challenging because sedation, analgesia, metabolic instability, temperature management, autonomic instability, and concomitant antiepileptic or sedative medications may both alter cortical excitability and obscure clinical signs of adverse events. Therefore, compared with prolonged DOC states, coma requires a more conservative, research-oriented safety posture, including conservative eligibility criteria, prespecified stop rules, continuous or repeated vital-sign monitoring, and EEG monitoring when feasible, particularly when clinical seizure signs may be suppressed or difficult to distinguish from the underlying acute illness.

In VS/UWS, patients usually cannot reliably report discomfort, headache, sensory symptoms, or subjective distress, so safety monitoring depends more heavily on external observation than on patient-reported tolerability. Adverse-event interpretation should therefore rely on structured observation, caregiver or nursing reports, changes in arousal, autonomic signs, sleep–wake disruption, behavioral fluctuation, and seizure surveillance. Because subtle behavioral changes may reflect spontaneous fluctuation rather than stimulation-related effects, safety and efficacy assessments should be separated whenever possible.

In MCS, more reproducible behavioral signs may allow better detection of discomfort, fatigue, agitation, or changes in arousal than in VS/UWS, but these same behavioral changes can also complicate interpretation of therapeutic response. Repeated-session protocols in MCS should therefore document cumulative exposure, session tolerability, coil stability, medication changes, sleep/arousal state, adverse events, and whether observed behavioral changes are sustained beyond immediate post-stimulation fluctuations.

### 6.3 Seizure risk and polypharmacy

While the absolute risk of major adverse events such as seizures remains low, careful screening is essential (Ripley et al., 2025). Risk factors such as intracranial metallic implants, a history of epilepsy, or recent intracranial bleeding must be evaluated case-by-case. This is especially critical when using high-frequency rTMS (HF-rTMS), which carries a slightly higher risk (Kim and Paik, 2021; Lefaucheur et al., 2020). Additionally, clinicians must account for polypharmacy—specifically drugs that lower the seizure threshold—which is common in DOC patients. Where feasible, documenting motor threshold and stimulation intensity, routine clinical observation (and EEG monitoring when available), adverse-event logging, and prespecified stop criteria can strengthen safety implementation and reporting. Seizure-risk management should also be interpreted in relation to DOC state and care setting, because available evidence does not yet support a simple subtype-based seizure-risk hierarchy. In coma, sedative and antiepileptic medications may suppress clinical seizure signs while also altering cortical excitability, making electrographic monitoring particularly important in exploratory studies. In VS/UWS, seizure-like events or subtle autonomic changes may be difficult to distinguish from baseline fluctuations. In MCS, patient movement, behavioral reactivity, agitation, or fatigue may affect coil stability and tolerability, and should be documented separately from clinically meaningful recovery.

### 6.4 Physiological heterogeneity

Finally, physiological responses to rTMS can differ across DOC subtypes. For example, increased local cerebral blood flow following stimulation has been reported in DOC, and hemodynamic responses may vary with both DOC subtype and stimulation parameters (Liu et al., 2016; Peng et al., 2025). This differential reactivity suggests that “responsiveness” is state-dependent and may also be modified by age, etiology, injury chronicity, and systemic physiological reserve, reinforcing the need for subtype- and population-specific monitoring rather than assuming that safety markers, tolerability indicators, or adverse-event detectability are identical across all levels of consciousness.

## 7 Discussion

The Discussion is organized according to four levels of inference: findings supported by current clinical evidence, biologically plausible but unproven mechanisms, limitations that constrain interpretation, and speculative directions for future study design. We first foreground the heterogeneity and unequal strength of the evidence and treat the rebooting/fine-tuning distinction only as a secondary, hypothesis-generating heuristic.

### 7.1 What is currently supported by clinical evidence

Current clinical evidence supports a cautious and provisional subtype gradient: rTMS shows the most consistent favorable signal in MCS, mixed findings in VS/UWS, and insufficient therapeutic evidence in coma. This conclusion is based on heterogeneous and unequal evidence rather than on equally powered subtype-specific trials. Therefore, the main evidence-based message is not that a validated treatment algorithm or framework-defined subtype strategy exists. Rather, future studies should move beyond average group effects toward patient stratification, mechanism-linked endpoints, and transparent reporting of key biological, clinical, access-related, and resource-level modifiers, such as age or developmental stage, etiology, injury chronicity, medication exposure, socioeconomic resources, caregiver support, geographic access, resource setting, and access to specialized neurocritical or neurorehabilitation services. Such clinical and methodological heterogeneity may influence apparent rTMS responsiveness not only by altering residual neuroplastic capacity, but also by shaping the metaplastic state that determines how the injured brain responds to repeated stimulation.

### 7.2 What is biologically plausible but not yet proven

In this subsection, biological plausibility refers to mechanisms and biomarker-linked interpretations that may help explain observed patterns but do not by themselves establish therapeutic efficacy. Plausible but unproven mechanisms include modulation of neurotransmitter homeostasis, BDNF-related synaptic plasticity, metaplastic thresholds, and large-scale network dynamics involving frontoparietal and thalamocortical circuits. These mechanisms may help explain why rTMS responsiveness appears to differ across DOC subtypes, but much of this evidence remains indirect, inferred from broader TMS literature, non-DOC populations, or preclinical models. Accordingly, mechanistic explanations such as synaptic potentiation, metaplastic gating, or network reintegration should be treated as biological plausibility rather than subtype-specific causal proof.

At the subtype level, this cautious interpretation suggests that MCS may provide a more plausible substrate for therapeutic rTMS testing because it is associated with more reproducible behavioral signs and more preserved network function. VS/UWS may involve more limited network readiness, whereas coma remains primarily a setting for residual-reactivity assessment and future exploratory study design. These distinctions are useful for hypothesis generation, but they should not be interpreted as evidence that different DOC subtypes already have validated stimulation mechanisms or clinically actionable protocols.

### 7.3 Inferential boundaries between clinical, biomarker, and mechanistic evidence

To separate what is clinically supported from what is biomarker-based or mechanistically inferred, we distinguish three levels of inference. First, diagnostic or probing evidence from TMS-EEG, EEG, neuroimaging, or MEP-based measures can reveal residual reactivity, connectivity, or corticospinal integrity. Second, therapeutic evidence requires clinically meaningful changes after repeated or patterned stimulation, such as CRS-R improvement, diagnostic transition, or sustained behavioral change. Third, mechanistic or intermediate biomarker changes after stimulation may support biological plausibility but should not be treated as therapeutic efficacy unless linked to clinical outcomes. These inferential boundaries also define the main limitations of the present narrative synthesis.

### 7.4 Limitations and constraints on inference

This review has several limitations. First, although we used a structured literature-search approach, this article remains a narrative review rather than a systematic review or meta-analysis. We did not perform PRISMA-based screening, formal risk-of-bias assessment, or quantitative pooling; therefore, selection bias in literature identification, prioritization, and interpretation cannot be excluded. Second, the therapeutic evidence base is limited by small samples, mixed DOC subtypes, case reports, uncontrolled or non-randomized designs, short follow-up periods, and variable outcome definitions. These limitations are particularly relevant when interpreting the apparent subtype gradient, because MCS, VS/UWS, and coma are not represented by equally powered or equally controlled studies. Third, stimulation protocols remain highly heterogeneous, including differences in target selection, localization method, frequency, intensity, pulse number, treatment duration, and combined interventions, which limits direct comparison across studies. In addition, target selection often reflects mixed mechanistic, historical, accessibility-related, and methodological considerations rather than prespecified, validated biological rules. Evidence for combination approaches is especially preliminary, and available reports generally cannot isolate the effects of rTMS from concomitant tDCS, DBS, pharmacological treatment, spontaneous recovery, or routine rehabilitation. Fourth, many mechanistic explanations, including LTP/LTD-like plasticity, glutamate/GABA modulation, dopamine release, BDNF-related effects, and large-scale network reorganization, are partly inferred from broader TMS literature, non-DOC populations, or preclinical models. Fifth, diagnostic uncertainty, fluctuating arousal, covert consciousness, and cognitive motor dissociation may influence baseline classification and apparent subtype-dependent responsiveness. Finally, incomplete reporting of population-, access-, and resource-related factors, including age distribution, pediatric versus adult status, sex, gender-related variables, race, ethnicity, socioeconomic resources, etiology, time since injury, comorbidities, medication exposure, caregiver support, and access to specialized neurocritical or rehabilitation services, may obscure treatment effects and limit generalizability. Therefore, both the apparent subtype gradient and the framework proposed here should be interpreted as hypothesis-generating rather than clinically prescriptive; biomarkers such as EEG, TMS-EEG, fMRI, PET, and connectivity measures should support diagnosis, prognosis, stratification, or mechanistic interpretation, but are not yet validated as standalone tools for treatment selection or parameter optimization in DOC.

### 7.5 What remains speculative and should guide future study design

This subsection addresses research directions rather than conclusions supported by current evidence. What remains speculative is not only the rebooting/fine-tuning distinction itself, but more importantly how any subtype-differentiated observation can be translated into reproducible treatment strategies. Frequency-dependent hypotheses—such as HF-rTMS for MCS potentiation or LF-rTMS for suppression in selected VS/UWS profiles—remain unproven and require robust confirmation. Future studies should therefore combine adequately powered, subtype-stratified RCTs with multi-institutional prospective registries and carefully designed non-randomized interventional studies. For causal interpretation, these studies should prespecify causal assumptions using directed acyclic graphs and systematically collect key confounders and effect modifiers, including age, etiology, injury chronicity, baseline CRS-R profile, sedation and medication exposure, comorbidities, socioeconomic resources, caregiver support, geographic access to specialized care, and resource setting. Such frameworks could guide target trial emulation, propensity score or inverse probability weighting approaches, sensitivity analyses for unmeasured confounding, and risk-of-bias assessment in non-randomized interventions (Hernán and Robins, 2016; Sterne et al., 2016).

Implementation-oriented tools are even more preliminary. AI-assisted technologies and machine-learning approaches may eventually help narrow the gap between conceptual neuromodulation frameworks and real-world practice. In DOC, such models could integrate CRS-R subdomain profiles, etiology, injury chronicity, medication exposure, EEG/TMS-EEG features, neuroimaging markers, stimulation parameters, and access-related variables to support diagnosis, prognosis, responder stratification, and hypothesis generation for candidate targets or protocol refinement. Importantly, AI should be used as a decision-support tool rather than a replacement for expert clinical assessment, and its use in DOC should require external validation, interpretability, bias auditing, and transparent reporting across diverse resource settings (Lee and Laureys, 2024). Because prediction models do not by themselves establish causal treatment effects, they should be complemented by causal-inference frameworks when evaluating whether rTMS changes clinical outcomes. Other exploratory directions should be treated with similar caution. Combination approaches, such as pairing rTMS with tDCS, DBS, or targeted neuropharmacology, remain highly preliminary in DOC. At present, they should be discussed as exploratory hypotheses for carefully controlled studies rather than as near-ready strategies for rational personalization. Future work should determine whether combined interventions provide additive or synergistic benefit, identify appropriate patient subgroups and timing, and establish safety before they are incorporated into individualized treatment frameworks. Future studies should also incorporate the state- and setting-dependent safety monitoring principles outlined above, particularly the differing seizure-surveillance needs, adverse-event detectability, and monitoring feasibility across coma, VS/UWS, and MCS. Future multicenter studies should prospectively capture biological, clinical, intersectoral, and resource-related variables to test whether rTMS effects are reproducible across diverse DOC populations and health systems. Collectively, these efforts may help determine whether rTMS can become an evidence-based component of neurorehabilitation for selected DOC populations, while maintaining a clear distinction between supported clinical signals, plausible biological mechanisms, and speculative implementation strategies.

## 8 Conclusion

Current evidence supports a cautious, hypothesis-generating view of state-dependent rTMS in DOC, with the most consistent clinical signal in MCS, heterogeneous findings in VS/UWS, and insufficient therapeutic evidence in coma. At present, evidence remains insufficient to guide patient-specific target or parameter selection for rTMS in routine clinical practice.

## Glossary

AMPA: *α*-Amino-3-hydroxy-5-methyl-4-isoxazolepropionic acid
BAEP: brainstem auditory evoked potential
BDNF: brain-derived neurotrophic factor
CGI-I: clinical global impression-improvement
CMCT: central motor conduction time
CRS-R: Coma Recovery Scale-Revised
DBS: deep brain stimulation
DLPFC: dorsolateral prefrontal cortex
DMN: default mode network
DOC: disorders of consciousness
dTMS: deep transcranial magnetic stimulation
ECN: executive control network
EEG: electroencephalogram
fMRI: functional magnetic resonance imaging
GABA: gamma-aminobutyric acid
GCS: Glasgow Coma Scale
Glu: Glutamate
HF-rTMS: high-frequency repetitive transcranial magnetic stimulation
iTBS: intermittent theta-burst stimulation
IPL: inferior parietal lobule
LF-rTMS: low-frequency repetitive transcranial magnetic stimulation
LTD: long-term depression
LTP: long-term potentiation
M1: primary motor cortex
MCS: minimally conscious state
MEP: motor evoked potential
NMDA: N-methyl-D-aspartate
PET: positron emission tomography
ppTMS: paired-pulse transcranial magnetic stimulation
PPC: posterior parietal cortex
PVS: persistent vegetative state
RCT: randomized controlled trial
RMT: resting motor threshold
rTMS: repetitive transcranial magnetic stimulation
sTMS: single-pulse transcranial magnetic stimulation
TBI: traumatic brain injury
tDCS: transcranial direct current stimulation
TEP: TMS-evoked potential
TMS: transcranial magnetic stimulation
TMS-EEG: transcranial magnetic stimulation combined with electroencephalography
UWS: unresponsive wakefulness syndrome
VS: vegetative state
CaMKII: calcium/calmodulin-dependent kinase II
SICI: short-interval intracortical inhibition
ICF: intracortical facilitation
cTBS: continuous theta-burst stimulation
DAN: dorsal attention network
